# Inherently Disordered Auxetic Metamaterials

**DOI:** 10.1002/advs.202521908

**Published:** 2026-03-19

**Authors:** Matteo Montanari, Reza Moghimimonfared, Andrea Spaggiari, Luke Mizzi

**Affiliations:** ^1^ Department of Sciences and Methods for Engineering University of Modena and Reggio Emilia Reggio Emilia Italy

**Keywords:** auxetics, delaunay triangulations, disorder, mechanical metamaterials

## Abstract

Inherently disordered auxetic systems represent a new class of metamaterial structures, which have the capability of exhibiting advanced functionalities despite the absence of global symmetry and ordered localized periodicity. In this work, disordered frameworks based on the Delaunay‐triangulation network incorporating chiral honeycomb features are proposed. These systems show large auxetic behavior, which is retained irrespective of the extent of disorder and depends entirely on the degree of chiralization. Furthermore, the Young's modulus of these systems can be controlled as a function of seed density, similar to how volume fraction determines stiffness in foams, without altering the auxeticity of these systems. Finally, the wide‐ranging parametric finite element study and experimental tests carried out in this work, revealed that the disordered topology of these systems significantly alters the failure pathways and deformation propagation profile of these metamaterials with respect to their ordered counterparts. The findings of this work clearly demonstrate that advanced functionalities such as auxeticity are not exclusive only to ordered frameworks and that, by harnessing disorder, one may potentially obtain additional superior properties, which are not present in traditional metamaterials.

## Graphical abstract

Inherently disordered auxetic metamaterials based on random chiral Delaunay triangulations are designed and investigated using numerical simulations and experimental tests. These disordered frameworks exhibit orthotropic behavior and a large negative Poisson's ratio (ca. −1), which is unaffected by seed density, while the stiffness can be freely modulated without changing the level of auxeticity. These random systems are also characterized by anomalous failure modes and deformation pathways under large strain loading.

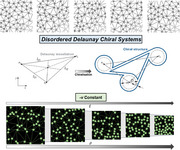

## 1 Introduction

Mechanical metamaterials [1, 2, 3, 4] are rationally designed materials with structure‐dependent counterintuitive mechanical properties, which are not found in naturally occurring materials. These anomalous properties include “negative” characteristics such as auxeticity (negative Poisson's ratio) [5, 6], negative stiffness [7, 8] and negative compressibility [9, 10]. These unusual characterstics can impart advanced functionalities and superior properties in comparison to conventional materials. Mechanical metamaterials are typically characterized by intricate and complex geometric motifs and the advent of rapid prototyping technologies such as additive manufacturing have enabled the physical realization of mechanical metamaterials at macro‐ and the microscopic scale. In particular, auxetic materials have attracted significant attention in recent years due to their exceptional properties for real‐world applications such as in impact/ballistic [11, 12], acoustic [13, 14], plasmonic [15, 16], actuation [17], aerospace [18, 19], and biomedical fields [20, 21, 22], just to mention a few.

In the scientific literature a vast number of geometries and mechanisms, which give rise to auxeticity may be found. These systems are subdivided into various classes on the basis of deformation mechanism, topology and symmetry and their mechanical properties may be tuned as a function of the geometric parameters. Yet, they share one common characteristic; they are characterized through a singular tessellatable building block, known as a representative volume element (RVE), which is replicated periodically in 2D or 3D space to form a superstructure, similar to how a repeating unit cell forms a pristine crystal lattice system at the nanolevel. This ordered framework ensures ease of design through predictable global deformation modes and a balanced distribution of localized stresses throughout the entire system. However, this also entails high sensitivity to defects since any imperfections within the system, which introduce local asymmetry may result in discontinuities that can have debilitating effect on the deformation mechanism, and hence, functionality of the metamaterial [23, 24, 25, 26, 27, 28], as well as the strain tolerance and failure mode. Although not particularly significant at the macroscale, this factor becomes a key limitation for fabrication of metamaterials at the lower micro‐ and nanolevels, where advanced additive and subtractive manufacturing techniques are pushed to their resolution and precision limits, leading to high defect concentrations.

The design of disordered metamaterials, which are functional despite their lack of symmetry and periodicity, could be a potential solution to this problem, since in such systems the influence of local geometric imperfections would be minimal on the global structure. It is worth noting that, although preferable, order is not a necessary precondition for auxeticity. Indeed, the first known example of a synthetic auxetic material is that of auxetic foam by Lakes [29], which is characterized by an inherently disordered microstructure. Disordered structures can have some advantages with respect to ordered ones. Studies on the fracture mechanics of nacre‐like structured materials have shown that the introduction of moderate levels of randomness can increase toughness due to crack deflection [30]. Similar findings were also observed from disordered lattice‐based stretch‐dominated structures [31]. More specifically, in the field of auxetic metamaterials, studies on re‐entrant hexagonal honeycombs subjected to topologically controlled levels of disorder analyzed in terms of fracture behavior have shown that the incorporation of disorder can be used to alter and improve the fracture mechanics of the system [32]. In terms of elastic properties, studies on the influence of translational disorder in hexachiral honeycombs have also shown that the introduction of spatial randomness can induce a significant increase in stiffness in comparison to the corresponding pristine systems [33], while other works have also indicated that intrinsically disordered networks can exhibit auxeticity at a comparable level to ordered systems [34, 35, 36, 37, 38, 39, 40]. These studies highlight the promising nature of this emerging branch in mechanical metamaterials research, and are clear evidence that not all forms of disorder are detrimental to metamaterial performance (as amply demonstrated for certain random and defected configurations by many works [23, 24, 25, 26, 27, 28]). They also demonstrate that by harnessing disorder in metamaterials in the correct manner, it may be possible to obtain amorphous systems, which can exhibit a similar and, in some cases, even superior level of mechanical performance compared to ordered lattice structures.

In view of this, we have designed a novel inherently disordered auxetic metamaterial system based on a randomly generated Delaunay triangulation‐based chiral network. Unlike typical studies on disordered metamaterials found in the state‐of‐the‐art, where a functional pristine auxetic network is rendered “disordered” through geometric perturbations, which are gradually inserted by random spatial translation of components or arbitrary removal/addition of substructures, we have departed directly from an inherently disordered network. This disordered network, that is, the Delaunay‐triangulation [41] generated from randomly placed points in 2D space, is then transformed into an inherently disordered auxetic system through a “chiralisation” [42, 43] process—whereby circular nodes are formed at the vertices of the triangulation and connected together through tangentially attached ligaments. This technique has already been utilized by the same authors on ordered Euclidean tessellations to transform them into auxetic metamaterial systems from conventional structures (i.e., positive Poisson's ratio systems) and in this work, we apply it for the first time to disordered networks. This approach is inspired by reconversion techniques used to transform conventional foam into auxetic foam, which function by introducing geometric characteristics typical of auxeticity in the microstructure whilst retaining the original disordered topological framework [44, 45, 46]. We demonstrate in this work how Delaunay‐based disordered chiral systems, which are shown to exhibit high levels of effective stiffness and large negative Poisson's ratio behavior, present a viable route toward the design of inherently disordered auxetic networks, which are fully functional notwithstanding the complete absence of symmetry and long range lattice‐based order.

## 2 Concept and Design

In order to analyze the mechanical properties of these Delaunay tessellation‐based disordered chiral metamaterials, we simulated a wide range of systems using numerical methods. The first step was the generation of the Delaunay tessellation. This was carried out by randomly generating a number of points, henceforth referred to as seeds, within a fixed space limit. Given a cloud of seeds $S=\{P_{1},P_{2},\cdots ,P_{n}\}$ in $R^{2}$, the Delaunay triangulation T consists of triangles where, for any triangle $▵P_{i}P_{j}P_{k}$, a fourth seed $P_{m}\notin \left\{P_{i},P_{j},P_{k}\right\}$ satisfies the empty circumcircle criterion. Notably, a triangle $▵P_{i}P_{j}P_{k}$ with points $P_{i}=\left(x_{i},y_{i}\right)$, $P_{j}=\left(x_{j},y_{j}\right)$, and $P_{k}=\left(x_{k},y_{k}\right)$ satisfies the Delaunay condition if and only if, for any other point $P_{m}=\left(x_{m},y_{m}\right)$, the determinant

(1) $$\det\left(A\right)=\left|\begin{array}{cccc} x_{i} & y_{i} & x_{i}^{2}+y_{i}^{2} & 1\\ x_{j} & y_{j} & x_{j}^{2}+y_{j}^{2} & 1\\ x_{k} & y_{k} & x_{k}^{2}+y_{k}^{2} & 1\\ x_{m} & y_{m} & x_{m}^{2}+y_{m}^{2} & 1 \end{array}\right|$$

satisfies det(A)≤0. If det(A)>0, the point $P_{m}$ lies inside the circumcircle of $▵P_{i}P_{j}P_{k}$, violating the Delaunay condition. Thus, a valid Delaunay triangulation ensures that all determinant values for external points are nonpositive [41]. All Delaunay tessellations created through this method are completely random and unique, with each randomization process yielding a different structure.

For the purpose of this study, we produced periodic randomly generated Delaunay tessellations. Although the design of “disordered” periodic systems may seem at first glance to be an unorthodox and paradoxical approach, this decision was made on the basis of three reasons. The first is that the influence of edge effects needs to be eliminated in order to ensure that the resultant mechanical properties are determined solely by the internal composition of the disordered framework rather than boundary factors. The second is that by utilizing periodicity, the continuum mechanical properties of the systems can be obtained, allowing for the reconstruction of the compliance matrix and a complete elucidation of the mechanical behavio of these systems. Finally, the third reason is that, in theory, if a sufficiently large number of seeds is utilized, the internal disorder within the system is ample enough to ensure that homogeneity is achieved and thus, in spite of the presence of periodicity, the system will still deform as a locally disordered network with a homogeneous global behavior. This reasoning is analogous to that utilized to determine the correct supercell size in molecular dynamics simulations, where convergence is achieved when the number of RVEs making up the supercell is sufficiently large enough so as to ensure that all the random oscillations within the system yield a stable, replicable solution.

**FIGURE 1:**
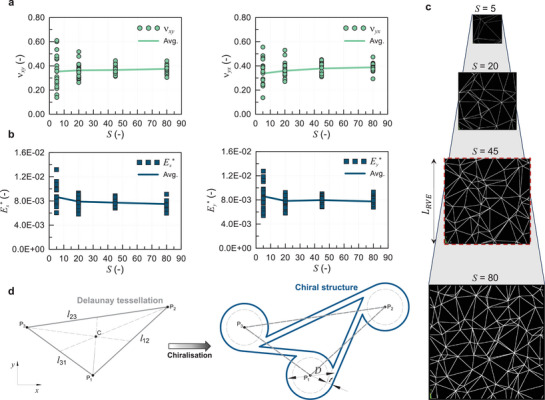
Trends of $\nu_{xy},\nu_{yx}$ (a) and $E_{x}^{*},E_{y}^{*}$ (b) for various combinations of seeds count and RVE size. View of the Delaunay tessellations with S=(5,20,45,80) (c). Schematic of the chiralization of the Delaunay triangulation (d).

In view of this, the next step was to determine, which is the minimum number of seeds that is required to achieve a sufficient level of homogeneity. Accordingly, we carried out disorder convergence testing directly on the Delaunay tessellations. Four sets, each with 20 samples, were generated with seed numbers, *S*, of 5, 20, 45, and 80. In order to ensure that the density of the system remained constant, the size of the square RVE (with side length $L_{RVE}$) was adjusted for each seed level to keep the seed density, ρ= $S/L_{\text{RVE}}^{2}$ constant at $2e^{-5}$ $mm^{-2}$ (i.e., the $L_{RVE}$ value was varied respectively for each *S* value as follows: 500, 1000, 1500, and 2000 mm). This seed density constant was chosen following extensive disorder convergence analyses, and as shown later on in this work, has no significant influence on the Poisson's ratio of the system provided that an appropriate number of seeds, *S*, is utilized. The Delaunay systems were built using beam elements with a rectangular cross‐section with an in‐plane thickness 2 mm and out‐of‐plane thickness of 1 mm and subjected to uniaxial loading in the x‐ and y‐directions separately under periodic boundary conditions (see Experimental section and Supporting Information for more details). The results obtained (shown in Figure 1a,b) clearly highlight that S = 45 is a suitable seed number to capture the influence of disorder on these systems with the Poisson's ratios equal to $\nu_{xy}=0.37\pm 0.03$ and $\nu_{yx}=0.38\pm 0.05$, and effective Young's moduli equal to $E_{x}^{*}=0.008\pm 0.0007$ and $E_{y}^{*}=0.008\pm 0.0007$, respectively. The two later parameters are dimensionless constants calculated by dividing the metamaterial Young's modulus in direction *i* with the constituent material isotropic Young's modulus.

Following the generation of the Delaunay tessellations, the systems were subjected to a “chiralization” geometric transformation. This was carried out by forming circular nodes at the vertices with diameter D, which are connected together through tangentially attached ligaments with thickness, t (see Figure 1). The extent of chiralization is controlled through these two parameters with configurations with large D/t ratios being considered as highly chiralised while, conversely, systems with small D/t ratio being the least transformed. In order to simulate these systems, PLANE elements under plane‐stress conditions were used and following mesh convergence analysis, a constant mesh size of t/3 was used.

A parametric simulation run was carried out on several disordered chiral systems with the optimal seed number (S = 45) with various D and t values. The influence of seed density on mechanical properties was also investigated and the results obtained are compared with corresponding ordered hexachiral honeycomb systems, which may be considered as an ordered variant of the random Delaunay triangulation. Finally, experimental tests on additively‐manufactured prototypes were conducted and analyzed.

## 3 Results and Discussion

**FIGURE 2:**
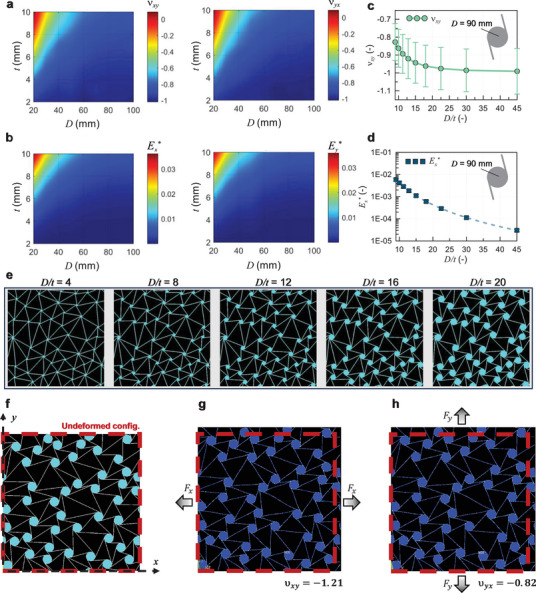
Influence of beams width t and chiral nodes diameters D on the elastic behavior of the chiral structures, in terms of Poisson's ratios $\nu_{xy},\nu_{yx}$ (a) and Young's moduli $E_{x},E_{y}$ (b). (a) and (b) represent the averaged values over 10 different geometries. (c) and (d) represent the trend for $\nu_{xy},\nu_{yx}$ and $E_{x},E_{y}$ for a geometry with D = 90 mm and varying t, respectively. (e) shows 5 chiral systems based on the same random Delaunay triangulation characterised by t = 5 mm and varying D from 20 to 100 mm. Here, all the different structures are characterized by S=45 and $L_{RVE}=1500$ mm. Deformed configurations under x‐loading (g) and y‐loading (h) are shown alongside the undeformed structure (f). The deformations are visualized using a magnified displacement scaling in order to enhance the visibility of the underlying deformation mechanisms.

The averaged results obtained from the chiralization of 10 periodic disordered Delaunay triangulation networks are presented in Figure 2a. The systems were each generated using 45 random seeds within an RVE of size 1500×1500 ${\mathrm{mm}}^{2}$. Ten configurations, corresponding to a total of 810 combinations of the geometrical parameters D and t – with t=(2,3,4,5,6,7,8,9,10) mm and D=(20,30,40,50,60,70,80,90,100) mm were carried out, with the largest D/t value being 100/2 and the lowest 20/10. These two limits characterize the most highly and least chiralized systems, respectively, with the latter being the most similar to the original ligament‐only‐based Delaunay tessellation.

It is evident from the plots that the chiralization process has successfully transformed the inherently disordered Delaunay systems into chiral metamaterials with a strongly negative Poisson's ratio. The most highly chiralized systems show Poisson's ratio of ca. −1, which is equal to that expected from an ordered hexachiral honeycomb system (i.e., the corresponding ordered version of these disordered chiral systems). This is a significant change from the original positive Poisson's ratio of +0.37 exhibited by the original non‐chiralized Delaunay networks. It is also clear that, even for systems with relatively small chiral node diameters, the transformation from non‐auxetic to auxetic systems is apparent immediately, indicating that chiralization is effective even at small levels. The presence of the chiral deformation mechanism; characterized by rotation of chiral nodes and flexure of ligaments is also evident from Figure 2g,h. This finding is congruent with those observed in previous studies on the chiralization of ordered triangular tessellations [47] and demonstrates how this geometric transformation procedure is equally effective on both ordered and disordered frameworks.

The trends obtained for the effective Young's modulus, $E^{*}$ also correspond to those typically obtained from ordered chiral metamaterials. The stiffness of these systems decreases as the pliant chiral deformation mechanism becomes more dominant with increasing node diameter, D, and decreasing ligament thickness, t. This marked drop in stiffness along with the drastic change in the Poisson's ratio as the extent of chiralization increases is indicative of the migration of the deformation mechanism from a stretch‐dominated original Delaunay framework to a rotation and ligament bending based chiral metamaterial system. This relatively high effective stiffness coupled with large auxetic behavior is also characteristic of chiralized ordered triangular tessellations and it appears as though these disordered systems exhibit analogous mechanical properties despite the lack of localized symmetry. This observation indicates that the topology (i.e., the triangulation framework) rather than group symmetry of the system is the governing factor, which determines the mechanical properties of these metamaterial networks.

In Figure 2c,d, the results for a subset of systems from the contour plots with D=90 mm with varying *t* values are plotted. The trends obtained from this subset are representative of the entire dataset and show how the effective Young's modulus varies according to a power law $E^{*}\propto {\left(D/t\right)}^{3}$, as t grows. The change in Poisson's ratio is also evident with the system becoming more auxetic as the D/t ratio increases, stabilizing fairly early at a Poisson's ratio between −0.9 and −1 from a D/t ratio of 15 onward.

At this point, it is also worth mentioning the small levels of anisotropy present in these systems. In theory, a random system is considered to be completely disordered if it exhibits a homogeneous response. From a mechanical point of view this translates to an isotropic system, or as in this specific case of 2D space, a transversely isotropic system. To evaluate the degree of anisotropy of our systems, we performed additional shear loading analyses and calculated a 2D elastic anisotropy index, $A^{\text{SU}}$, for ten representative structures (with D=100mm and t=5mm). Isotropic behaviour corresponds to $A^{\text{SU}}=0$, while the results obtained here yielded an average value of $A^{\text{SU}}=0.74\pm 0.11$. This analysis is based on the index proposed by Li et al. [48], who introduced $A^{\text{SU}}$ as a simple and universal measure of elastic anisotropy in 2D systems. The anisotropy index $A^{SU}$ is given by

(2) $$\begin{array}{ccc} A^{\text{SU}} & = & \left({\left[\frac{1}{4}\left(C_{11}+C_{22}+2C_{12}\right)\left(S_{11}+S_{22}+2S_{12}\right)-1\right]}^{2}\right.\\ & & {\left.+\;2{\left[\frac{1}{16}\left(C_{11}+C_{22}-2C_{12}+4C_{66}\right)\left(S_{11}+S_{22}-2S_{12}+S_{66}\right)-1\right]}^{2}\right)}^{\frac{1}{2}} \end{array}$$

For our sample set of ten representative structures (with D=100mm and t=5mm), we obtained an average value of $A^{\text{SU}}=0.73\pm 0.09$, indicating a moderate level of anisotropy in the considered geometries (more information on this can be found in the Supporting information). It is also worth noting that, in addition, all systems demonstrate complete in‐plane orthotropy, with the relationship $E_{x}/\nu_{xy}=E_{y}/\nu_{yx}$ being respected for all individual disordered structures simulated. Thus, the Poisson's ratio below −1 obtained from certain configurations such as the one shown in Figure 2g is accounted for by the orthotropy of the system and is accompanied by a corresponding Poisson's ratio above −1 in the transverse loading direction.

**FIGURE 3:**
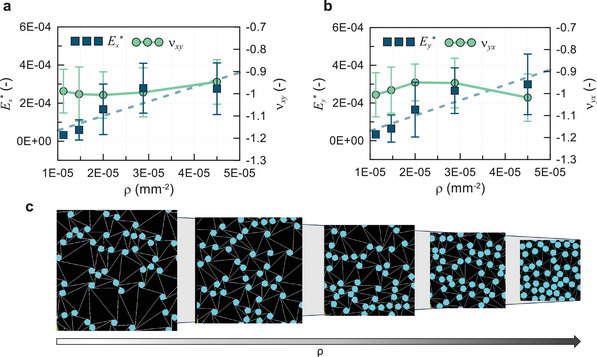
Influence of RVE size on the Young's moduli $E_{x}^{*}$ (a) and $E_{y}^{*}$ (b) and on the Poisson's ratios $\nu_{xy}$ (a) and $\nu_{yx}$ (b), respectively (the dashed blue lines represent a linear fitting of the numerical results). Delaunay chiral structures for different RVE sizes (c). Here, all the different structures are characterized by D/t=50.

The analysis of the influence of seed density on mechanical properties also yielded extremely interesting results. While keeping the number of seeds constant at S=45 (and D/t = 50), the dimension of the representative volume element (RVE) was varied. Specifically, five different cases were considered: $L_{RVE}=\left(1000,1250,1500,1750,2000\right)$ mm with 10 samples each, as shown in Figure 3a,b. These values correspond to seed densities, ρ, of $4.5e^{-5}$, $2.88e^{-5}$, $2e^{-5}$, $1.47e^{-5}$, and $1.13e^{-5}$ $mm^{-2}$, respectively. The results obtained clearly show that the densification of the system is followed by a *quasi*‐linear increase in stiffness. This is consistent with the behavior of ordered chiral honeycombs with limited degrees of freedom such as hexachiral honeycombs, which deform primarily via pure flexural deformation of ligaments and rotation of chiral nodes. Remarkably, the Poisson's ratio remains almost unchanged, at a constant range between −0.9 and −1, regardless of the increase in point density, which is also a characteristic of ordered systems. This is highly noteworthy since it demonstrates that despite the randomness and lack of symmetry, which characterizes these systems, their linear mechanical properties and deformation behavior are exceedingly similar to those of ordered auxetic metamaterials. This finding also leads to the conclusion that one may vary the stiffness of these disordered frameworks as a function of volume fraction without compromising the auxeticity of the system. This is extremely significant, since in typical disordered realistic auxetic systems such as foams, variation of stiffness without a change in Poisson's ratio is not achievable, that is, in auxetic foams, an increase in density is typically accompanied by an increase in Young's modulus and a reduction in auxeticity [49, 50]. On the other hand, the chiralized disordered Delaunay systems presented in this work allow for tuneability of stiffness independent of Poisson's ratio, which is a rare property.

In order to validate and confirm these findings, five sample disordered Delaunay systems were fabricated using additive manufacturing along with a corresponding regular hexachiral [51] honeycomb system. The latter may be considered to be the closest “ordered” equivalent of the disordered systems studied in this work and thus presents an ideal example for comparative purposes. All systems were subjected to uniaxial *quasi*‐static compressive loading and examined using image analysis techniques. Analogous nonlinear finite element simulations were also carried out on these structures under finite loading conditions. Full details on the methodology used are provided in the Experimental Section and Suppporting Information.

**FIGURE 4:**
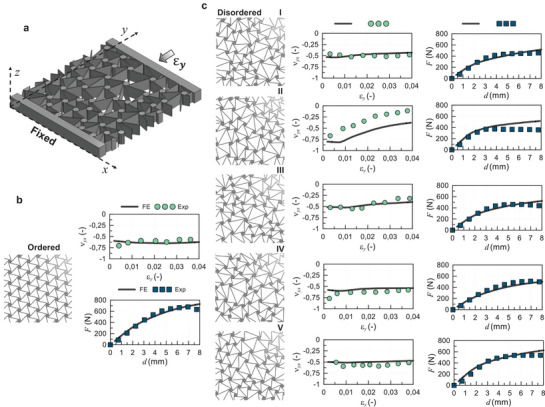
3D Schematic of the experimental setup (a). Comparison between the numerical and experimental Poisson's ratio $\nu_{yx}$ and force F up until a global applied deformation of ε=0.04 (corresponding to a displacement d=8 mm) for the hexachiral structure (b) and for the five representative disordered systems (c). The full force‐displacement graphs for strains up to 0.2 are presented in the Supporting Information.

As shown in Figure 4, all systems exhibited a negative Poisson's ratio, which was retained over a 4% compressive strain range. The ordered hexachiral system showed a *quasi*‐constant Poisson's ratio of ca. −0.6, while the disordered systems exhibited a similar trend, with a constant Poisson's ratio between −0.7 and −0.45 in the majority of cases. One system, shown in Figure 4c, demonstrated a relatively more negative initial Poisson's ratio of −0.72 at the beginning, which experienced a gradual reduction in magnitude with increasing compressive strain down to −0.2. This trend was only observed in this particular case; however, indicating that this is an anomalous characteristic, which is specific to this particular configuration. At this point, it is also worth noting that in all cases, including the ordered hexachiral system, the observed Poisson's ratio is slightly lower in magnitude than that obtained from simulations under periodic boundary conditions. This factor can be attributed entirely to edge effects, which are well known in the field of chiral metamaterials to result in a slight decrease in the auxeticity of these systems when finite systems with a relatively small number of RVEs are tested. This is confirmed by the fact that, as shown in Figure 4, the predictions of the nonlinear geometric simulations carried out under finite loading conditions analogous to the experimental testing mode used yielded almost completely matching results for both Poisson's ratio and force‐displacement behavior. In terms of stiffness, it can also be observed that the disordered systems all exhibited a similar stiffness constant and force‐displacement profile to the ordered hexachiral system, albeit, at a slightly lower value. These findings confirm that the influence of disorder and symmetry breakage in these systems is minimal on the auxetic properties and mechanical performance of these chiralzsed disordered networks.

**FIGURE 5:**
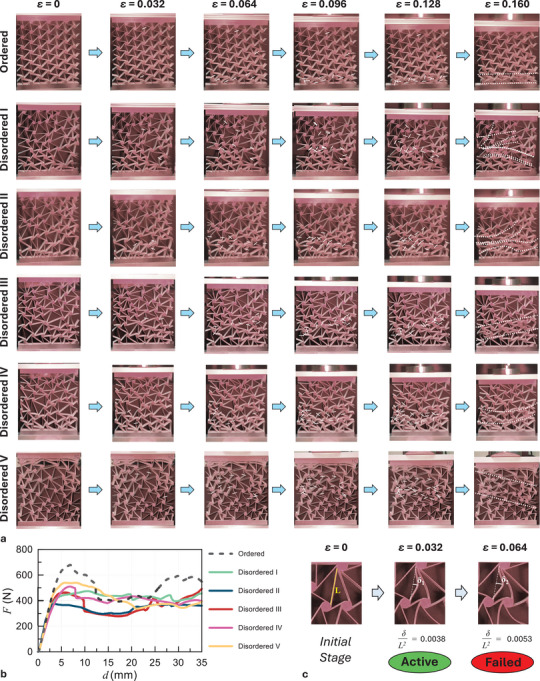
Images showing the failure modes upon increasing compressive strains for the experimentally tested additively manufactured ordered hexachiral honeycomb and all five disordered systems with the same seed density. The failed ligaments are indicated by white lines, with the overall pathway shown in the final panel at ε = 0.16 (a). The force‐displacement plots of these systems for the same strain range are shown as well (b). Panel (c) shows an example of a ligament from Disordered I, which was measured to gauge the level of deflection. The deflection to length squared ratio does not meet the 0.004 ${\mathrm{mm}}^{-1}$ threshold level at a strain of 0.032, hence it is still active, while at a strain of 0.064, this value was exceeded and hence the ligament was marked with a white line as failed.

In addition to the small strain evaluation, a high‐strain failure mode analysis was also carried out on these systems. The ordered and disordered systems were each subjected to compressive loads of up to 16% and failure modes of these systems were analyzed, as shown in Figure 5a, where the results obtained from the ordered hexachiral system and five disordered systems (Disordered I–V) are illustrated. In this work, we defined failure as the localized buckling/irreversible plastic yielding of ligaments, following the definition utilized by Ashby to describe failure modes of cellular solids [52]. This definition is also widely used in the literature to describe the compressive failure of ligament‐based cellular metamaterials, since fracture is rarely observed in these cases [53, 54, 55, 56]. In order to determine whether a ligament has underwent irreversible yielding, the deflections of the ligaments in the ordered and disordered systems were measured utilizing the images from the compressive loading tests. These deflections were then evaluated using the following failure criterion:

(3) $$\frac{\delta }{L^{2}}=0.004\;{\text{mm}}^{-1}.$$

This ratio was obtained by considering the stiffness and ultimate tensile strength of the constituent PLA material and applying the Euler–Bernoulli beam mechanics for C‐shaped flexure, which was the main deformation mode observed. A detailed explanation on how this criterion was calculated is provided in the Supporting Information.

It is clearly evident that the disordered systems exhibit completely different failure modes with respect to the ordered hexachiral system. While the latter, as expected, exhibits an ordered, predictable layer‐wise collapse [57, 58], which occurs almost instantaneously at a strain of about 3.5% (*d* = 7 mm), the disordered systems each exhibit a more peculiar failure pathway. In the case of Disordered I and II, rather than collapsing layer‐by‐layer, the longer ligaments near the edges start to gradually fail with the failure pathway slowly evolving in an inclined path toward other ligaments with increasing strain. This results in a longer, more gradual edge‐to‐edge pathway, which divides into more than one failure “veins.” In Disordered I and II, the failure pathway forks into two distinct branches. It is worth noting that both these disordered systems also do not suffer from the sudden whole‐layer collapse, which characterizes the failure profile of the corresponding ordered system, with the force‐displacement plots showing a plateau after the linear region rather than a negative slope, highlighting the gradual failure modes of these amorphous networks. On the other hand, in the Ordered system, the negative slope region which characterizes the whole‐layer collapse is clearly visible. In Disordered III–V, the systems exhibit force‐displacement behavior and failure propagation pathways, which are halfway between the Ordered and Disordered I and II systems. In these cases, the failure of ligaments is observed to propagate in a single pathway (with no visible forking as in Disordered I and II), which is inclined, and, therefore, longer than that of the ordered system, which evolves horizontally. This behavior is also reflected in the force‐displacement plots of these systems, which show a smaller, less distinct negative slope region than the corresponding ordered system. It is also worth noting that the extended plateau behavior of the disordered systems comes at a cost, since these systems plateau at a lower force and, as a result, exhibit significantly lower energy absorption potential.

These observations are extremely significant, since they are congruent with what has been observed in the literature on disordered and aperiodic non‐auxetic systems [30, 31, 59]. Although these studies deal primarily with fracture propagation rather than failure pathways, they also indicate that the pathway is altered significantly upon the introduction of disorder—with a longer route observed with respect to corresponding ordered systems. The systems investigated in these works found in the literature are all stretch‐dominated architectures and, thus, the similarity in failure pathways with the Delaunay‐chiral systems is remarkable, since besides being auxetic, the disordered systems studied in this work are not classified as stretch‐dominated geometry in terms of the classical Maxwell criterion [52, 60, 61]. The chiralisation of the Delaunay tessellation introduces additional degrees of freedom (hence transforming it into a bending‐dominated system); however, previous studies on ordered chiralized systems have shown that these systems still retain some vestiges of their previous form despite this geometric transformation such as *quasi*‐uniform distribution of localized deformations within ligaments. It appears that this factor also allows for the retention of other stretch‐dominated lattice characteristics upon chiralization besides topology, such as anomalous failure propagation pathways. Naturally, given the relatively small number of chiral nodes present in the experimentally tested samples of this work, we cannot quantitatively characterize this effect and further studies on much larger systems are required. Nevertheless, the fact that considerably different failure modes were observed in such small systems once comparing corresponding ordered and disordered chiral networks, suggests that this factor will be significantly amplified in larger finite systems. Further work needs to be carried out in order to quantitatively characterize the failure propagation pathways of these disordered auxetic systems utilizing larger representative sample sizes and significantly greater number of exemplars.

Before concluding, it is worth highlighting the significance of this work. The disordered Delaunay‐based chiral metamaterials proposed in this work represent a prime example of an inherently disordered metamaterial framework where auxeticity does not vary as a function of the level of disorder. This means that one can produce a random auxetic system where the stiffness is tailored as a function of density/volume fraction whilst retaining a highly negative Poisson's ratio of −1. This threshold value has not been achieved in disordered cellular systems such as auxetic foams which typically exhibit lower magnitude Poisson's ratio, which do not remain unchanged as density increases. The extent of auxetictiy in the disordered architectures proposed in this work can also be tailored according to the degree of chiralization. This level of control of mechanical properties is not a common feature of disordered architectures and, from this point of view, these Delaunay‐tessellation‐based disordered chiral metamaterials stand out as a particularly remarkable case. The robustness of these systems arises primarily from their triangular configuration, which results in the systems retaining the underlying topological features despite the introduction of disorder. In other classes of “spatially” or “translationally” disordered auxetic systems found in the literature based on higher *n*‐sided polygonal topological frameworks such as anti‐tetrachiral and re‐entrant hexagonal honeycombs, high levels of randomness typically alter the topology of the system resulting in a change of deformation mechanism leading to a decrease of auxeticity. Furthermore, the disordered framework of these chiral metamaterials has also been shown to impart anomalous failure propagation pathways and resistance to sequential layer‐by‐layer collapse similarly to non‐auxetic random cellular systems. The simultaneous achievement of large auxeticity with elongated, non‐uniform failure pathways in these disordered metamaterials is distinctive and demonstrates that even inherently disordered frameworks can be suitable topological candidates for the design of auxetic metamaterials with enhanced functionalities.

This finding also opens up several interesting avenues for further utilisation of these systems. The traditional Delaunay‐triangulation is an established method for the generation of meshes and infills in additive manufacturing and can theoretically incorporated into any closed‐form 2D shape. Since these chiralized Delaunay‐based systems can exhibit auxeticity even without symmetric/periodic arrangements, the use of infills based on these chiral architectures can be used to impart auxeticity to additively manufactured components, even in cases where the geometric complexity does not allow for the generation of uniform triangles. Moreover, the anomalous failure modes and deformation propagation observed for these disordered systems, could also be an additional advantage, since besides allowing for auxetic behavior, infills based on disordered Delaunay‐triangulation chirals could also potentially confer improved resistance against sequential layer‐wise collapse. Finally, the inherently disordered framework of these systems could also make them ideal candidates for realization at the lower micro‐ and nanolevel through random‐based self‐assembly methods, which typically yield “correlated‐disorder”[62] based networks, which lack global symmetry and a clearly defined representative unit cell. We hope that the findings of this work will help unlock the untapped potential of inherently disordered auxetic metamaterials and bring us closer to understanding the fundamental relationship between symmetry and functionality in mechanical metamaterials.

## 4 Conclusion

In this work, a new class of inherently disordered auxetic structures based on the Delaunay triangulation was proposed and thoroughly investigated through an extensive numerical and experimental approach. The results obtained showed that despite the lack of localized symmetry and short‐range order, these chiral metamaterials can exhibit a large negative Poisson's ratio of −1. The extent of auxeticity was shown to remain unchanged upon altering the seed density of the system, which means that one may vary the stiffness and volume fraction of the system without affecting the Poisson's ratio of the structure. Experimental tests on additively manufactured prototypes confirmed the findings obtained from the numerical simulations and also indicated anomalous failure modes and deformation propagation pathways in these systems, which are not typically found in ordered metamaterials. Overall, the results presented in this study prove that achieving auxetic behavior does not always require high symmetry and pristine geometries and that even disordered structures—generated through simple procedures and without strict geometric control—can effectively exhibit auxeticity, making disordered chiral Delaunay triangulation based metamaterials highly suitable for practical applications and large‐scale manufacturing.

## 5 Experimental Section

*Finite Element Simulations*: Three sets of FEM simulations were carried out. The first set involved simulations on traditional Delaunay‐tessellations made from BEAM elements in order to determine the optimal number of seeds to use for the parametric studies. The simulations were carried out on 20 samples of periodic frameworks with various representative volume element sizes/seed densities under periodic boundary conditions (as described in further detail in the Supporting Information) and the Poisson's ratios and Young's moduli in the principal axes were extracted. Once a seed size of 45 was obtained, a more wide‐ranging simulation run was carried out using PLANE elements on 10 disordered frameworks at various levels of chiralization through variations of the geometrical parameters *D* and *t*—with *t* = (2, 3, 4, 5, 6, 7, 8, 9, 10) mm and *D* = (20, 30, 40, 50, 60, 70, 80, 90, 100). These simulations were also carried out under periodic boundary conditions [63] under linear static loading conditions. Finally, five samples were chosen for the experimental testing, and these structures were simulated under nonlinear geometric loading conditions under finite conditions (similar to experimental compressive loading tests) in order to simulate their behaviour at strains of up to 5%. In all cases, the linear isotropic material properties of Polylactic Acid (PLA) were used with a Young's modulus of 2.2 GPa and a Poisson's ratio of 0.3.

*Mechanical Testing*: To validate the FEM simulations and support the findings presented in the preceding Sections, displacement‐controlled uniaxial compression tests were performed on five representative disordered chiral structures and one ordered hexachiral system manufactured via Fused Deposition Method (FDM). The specimens were fabricated using a Bambu Lab X1 Carbon 3D printer with PLA filament. Printing parameters include a 100% infill density with a 45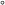/–45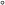 raster pattern and a layer height of 0.2 mm. The out‐of‐plane thickness of the printed samples was set to 20 mm. The systems each contained 45 seeds, with ligament thickness equal to 1 mm and node diameter of 10 mm. The guage length of each system was equal to 202 mm. Mechanical testing was carried out using a Galdabini Sun 500 universal testing machine equipped with a 5 kN load cell. A global compressive displacement of 40 mm (corresponding to approximately 20% strain along the *y*‐axis) was applied at a constant rate of 5 mm/min (Figure 4a) shows a schematic view of the experimental setup). During testing, videos are recorded using a 4K camera at a resolution of 3840 × 2160 pixels.

*Poisson's Ratio Measurement and Failure Mode Analysis*: The Poisson's ratio $\nu_{yx}$ was evaluated using a point tracking method, in which transverse strains $\varepsilon_{x}$ are computed based on the displacements of tracking points positioned along the left and right edges of the specimen, while the longitudinal strain $\varepsilon_{y}$ is derived from the displacement of the loading plate. A number of representative frames were extracted from the recordings at different stages of deformation subsequently used to measure the Poisson's ratio by tracking the horizontal and vertical displacements of the external borders of the structure over a strain range of 4% as shown in the Supporting Information. For the failure mode analysis, a wide strain range was considered of up to 16%. Through a visual analysis, ligaments showing large deflection were marked and used to determine the failure pathway of the overall system. Further details are provided in the Supporting Information section.

## Conflicts of Interest

The authors declare no conflicts of interest.

## Supporting information


**Supporting File**: advs74487‐sup‐0001‐SuppMat.pdf.

## Data Availability

The data that support the findings of this study are available in the supplementary material of this article.
