# Supplementary material for: Inherently Disordered Auxetic Metamaterials
Source: Adv Sci (Weinh). 2026 Mar 19;13(30):e21908. doi: 10.1002/advs.202521908 (PMC13248855; doi:10.1002/advs.202521908)
Supplement: Supplementary file 1 — Supporting File: advs74487‐sup‐0001‐SuppMat.pdf. [file ADVS-13-e21908-s001.pdf]

Supporting Information for Inherently disordered auxetic metamaterials

*Matteo Montanari Reza Moghimimonfared Andrea Spaggiari Luke Mizzi**

Dr. M. Montanari, Mr. R. Moghimimonfared, Prof. A. Spaggiari, Prof. L. Mizzi
Department of Sciences and Methods for Engineering, University of Modena and Reggio Emilia, Reggio Emilia, Italy
luke.mizzi@unimore.it

1 Construction of Periodic Delaunay Tessellations

In order to generate a periodic structure based on a disordered Delaunay tessellation, we begin by defining the initial set of S seed points within a fixed 2D space. The seed points are randomly generated using a continuous uniform distribution (Fig. S1a) and the fixed space will eventually form the representative volume element (RVE) of the disordered network. Following the initial point generation, the S seeds are replicated and translated around the RVE to form a 3×3 expanded supercell cell (Fig. S1b). Then, as shown in (Fig. S1c-d), the Delaunay triangulation is enacted using all $9S$ seeds through the condition defined in the matrix shown in Eq. 1 of the main manuscript. Once the Delaunay triangulation is formed, the central RVE of points, which corresponds to the original unit cell, is obtained by trimming away the regions outside the RVE boundaries. This approach allows to obtain a periodic RVE, which can be subjected to periodic boundary conditions in order to eliminate edge effects. The example shown in Fig. S1 represents a BEAM element system, however, the same approach was also utilised for the 2D PLANE element systems.

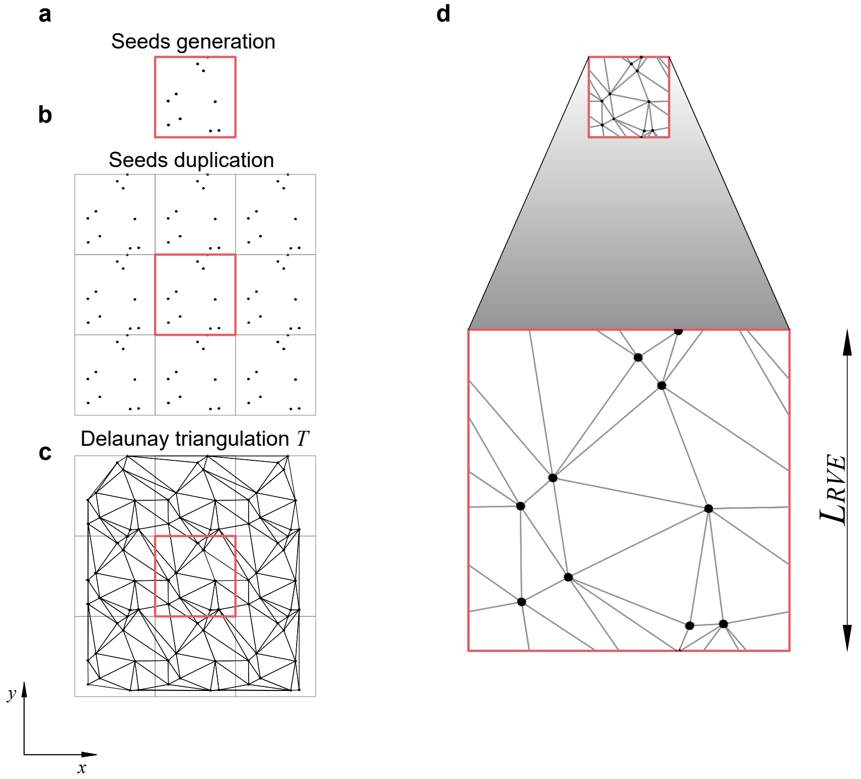

Fig. S1: Construction of a periodic Delaunay representative volume element (a-d). Schematic of the chiralisation of the Delaunay triangulation (e). The relatively low number of initial seeds is here chosen for the sake of visual clarity.

2 Periodic Boundary Conditions

In order to evaluate the effective mechanical properties of the tessellated structures, periodic boundary conditions (PBCs) are implemented on the representative volume element (RVE) to replicate the response of an infinite periodic medium. These conditions are imposed by enforcing constraint equations that govern the displacement and rotational degrees of freedom of the boundary nodes [1]. This complex and generalized approach is necessary due to the fact that these disordered systems possess no axes of mirror or rotational symmetry.

The formulation presented here applies to BEAM element simulations employing Timoshenko beam theory, where each node has six degrees of freedom: three translational components (u_x, u_y, u_z) and three rotational components $(\theta_x, \theta_y, \theta_z)$. This represents the most general case. Let u_x and u_y denote the in-plane displacements in the x- and y-directions, respectively, and let θ_z represent the rotation about the out-of-plane axis. Opposing boundary nodes are indexed by superscripts $i = (1, 2, 3, 4)$, representing two pairs of corresponding nodes on opposite sides of the RVE. The periodicity constraints are then expressed as follows:

$$u_x^{(1)} - u_x^{(2)} = u_x^{(3)} - u_x^{(4)} \tag{S1}$$

$$u_y^{(1)} - u_y^{(2)} = u_y^{(3)} - u_y^{(4)} \tag{S2}$$

$$\theta_z^{(1)} = \theta_z^{(2)} \tag{S3}$$

To ensure planarity of the two-dimensional model, all out-of-plane degrees of freedom are constrained at every node:

$$u_z = 0 \tag{S4}$$

$$\theta_x = 0 \tag{S5}$$

$$\theta_y = 0 \tag{S6}$$

To eliminate rigid body motion, one node located on the bottom edge of the RVE is fully fixed, while the corresponding node on the top edge is constrained in the x-direction only. This configuration ensures vertical alignment of the unit cell during deformation while permitting axial extension or contraction as well as angular shear-based deformation if required (see Figure S2).

In contrast, when simulations are performed on geometries discretized using 2D PLANE elements such as the chiralised disordered systems, which possess only u_x and u_y degrees of freedom, the formulation simplifies significantly as Equations S3–S6 become unnecessary, with only Equations S1 and S2 remaining active.

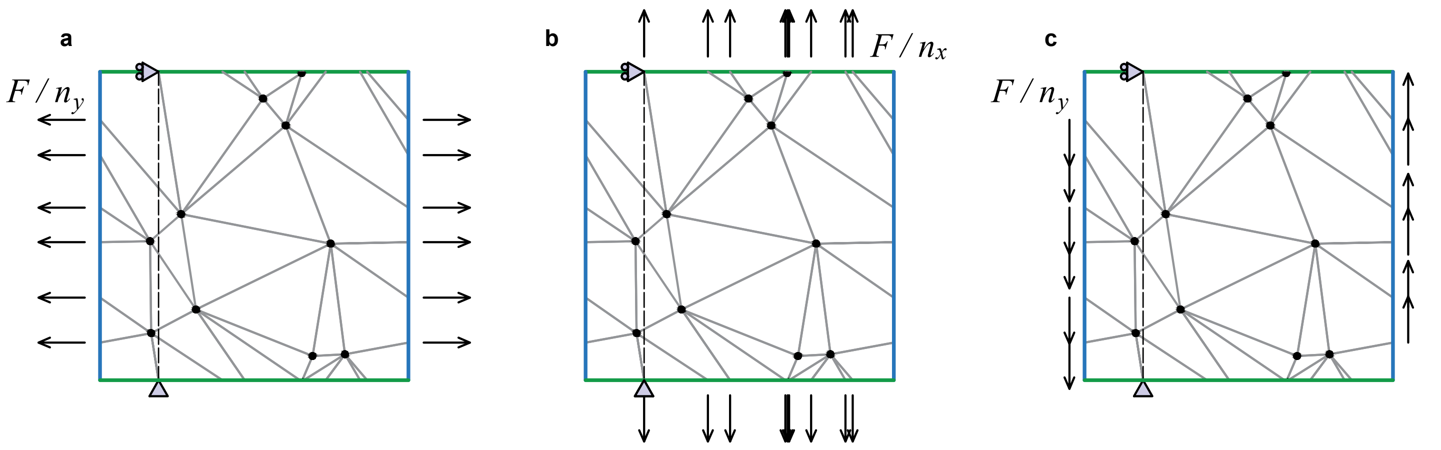

Fig. S2: Periodic boundary conditions for evaluating E_x, ν_{xy} (a), E_y, ν_{yx} (b), and G_{xy}, respectively.

In order to induce uniaxial loading of the disordered systems in the x- and y-directions, a uni-directional normal force was applied which was distributed equally over all the boundary nodes of the system as shown in Figure S2. On the other hand, when carrying out the shear loading simulations, the forces were applied parallel to the edges. More details on PBCs methodology employed in this work and its validation may be found in [1].

3 Elastic Anisotropy Measure

In order to confirm that the disordered architectures studied in this work exhibit in-plane orthotropy, the on-axis Poisson's ratios and Young's moduli extracted from the FE simulations were subjected to the reciprocity condition, indicated below:

$$\frac{E_x}{\nu_{xy}} = \frac{E_y}{\nu_{yx}} \tag{S7}$$

In Fig. S3 the results obtained for the disordered chiral systems are plotted. It is clearly evident that all systems are orthotropic, falling exactly on the equality threshold. This finding confirms the orthotropy verification and allows us to proceed to evaluate the level of anisotropy of these systems under the assumption of orthotropic behaviour.

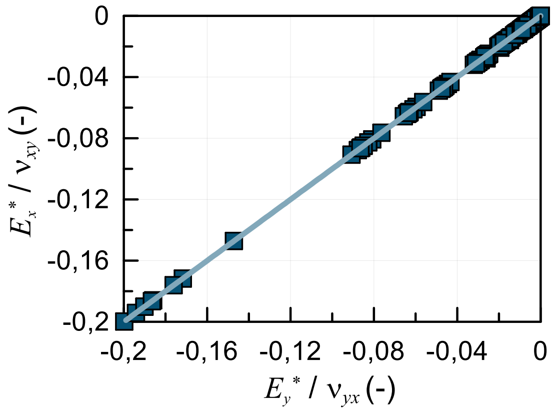

Fig. S3: Validation of the orthotropic behavior, for all the different chiral structures analyzed.

To assess the degree of elastic anisotropy in these architectures, the two-dimensional anisotropy index A^{SU} proposed by Li *et al.* [2] was utilised. This index is based on the in-plane stiffness matrix $\mathbf{C}$ and its inverse, the compliance matrix $\mathbf{S} = \mathbf{C}^{-1}$, defined in Voigt notation as follows:

$$\mathbf{C} = \begin{bmatrix} C_{11} & C_{12} & 0 \\ C_{12} & C_{22} & 0 \\ 0 & 0 & C_{66} \end{bmatrix} \tag{S8}$$

$$\mathbf{S} = \begin{bmatrix} S_{11} & S_{12} & 0 \\ S_{12} & S_{22} & 0 \\ 0 & 0 & S_{66} \end{bmatrix} \tag{S9}$$

For plane stress conditions and orthotropic behavior, the components of the compliance matrix can be expressed in terms of engineering constants as:

$$S_{11} = \frac{1}{E_x}, \quad S_{22} = \frac{1}{E_y}, \quad S_{12} = -\frac{\nu_{xy}}{E_x} = -\frac{\nu_{yx}}{E_y}, \quad S_{66} = \frac{1}{G_{xy}}. \tag{S10}$$

From these, the stiffness components can be obtained as:

$$C_{11} = \frac{E_x}{1-\nu_{xy}\nu_{yx}}, \quad C_{22} = \frac{E_y}{1-\nu_{xy}\nu_{yx}}, \quad C_{12} = \frac{\nu_{xy}E_y}{1-\nu_{xy}\nu_{yx}} = \frac{\nu_{yx}E_x}{1-\nu_{xy}\nu_{yx}}, \quad C_{66} = G_{xy}. \tag{S11}$$

The anisotropy index A^{SU} is given by:

$$A^{\mathrm{SU}} = \left(\left[\frac{1}{4}(C_{11}+C_{22}+2C_{12})(S_{11}+S_{22}+2S_{12}) - 1 \right]^2 + 2\left[\frac{1}{16}(C_{11}+C_{22}-2C_{12}+4C_{66})(S_{11}+S_{22}-2S_{12}+S_{66}) - 1 \right]^2 \right)^{\frac{1}{2}} \tag{S12}$$

A value of $A^{\mathrm{SU}} = 0$ indicates isotropic behavior, while larger values reflect increasing elastic anisotropy. For our sample set of ten representative structures (with $D = 100\,\mathrm{mm}$ and $t = 5\,\mathrm{mm}$), we obtained an average value of $A^{\mathrm{SU}} = 0.73 \pm 0.09$, indicating a moderate level of anisotropy in the considered geometries.

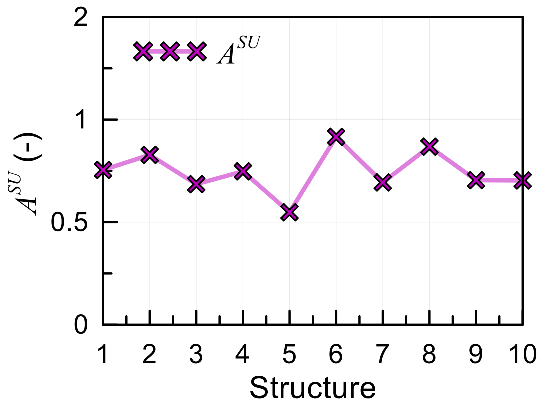

Fig. S4: Anisotropy index A^{SU} for the different structures considered ($D = 100$ mm and $t = 5$ mm).

4 Mesh Convergence

A preliminary mesh convergence study is performed on a disordered chiral system with $D = 80\,\text{mm}$ and $t = 4\,\text{mm}$, by varying the mesh size from 0.5 to 2 mm, which corresponds to 8 or 2 elements across the thickness t of the ligaments. The results of the finite element simulations indicate negligible differences across the range of mesh sizes considered.

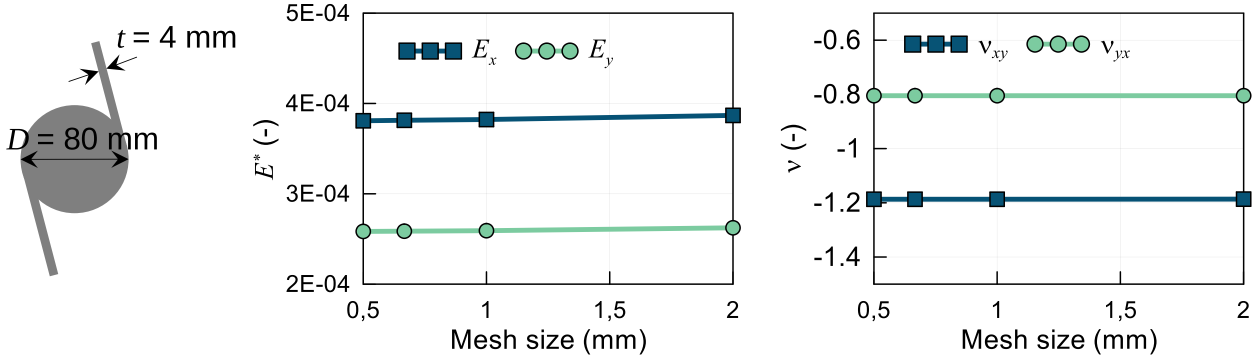

Fig. S5: Mesh convergence analysis, performed on a chiral system with $D = 80$ mm and $t = 4$ mm.

5 Influence of Ligament Length Dispersity

The influence of dispersity, generated as a result of disorder, is examined. In particular, in order to quantify the dispersity of the systems, we compute the length l of each side of all the Delaunay triangles from which the chiral structure is generated, and evaluate its standard deviation $\delta = \frac{\sqrt{\langle l^2 \rangle - \langle l \rangle^2}}{\langle l \rangle}$, where $\langle l \rangle$ is the mean of l.

It is found that the dispersity parameter δ increases linearly with the dimension of the representative volume element L_{rve} (see Figure S6a). This trend was to be expected since larger unit cells provide more free space, hence, a greater level of possible disorder, and thus by keeping the number of seeds constant, a greater dispersion in the element lengths will occur. Figures S6b-e show the elastic moduli E_x^* and E_y^*, as well as the Poisson's ratios ν_{xy} and ν_{yx} for a set of ten structures with a representative volume element (RVE) size of $L_{RVE} = 2000\,\text{mm}$, which exhibits the highest average dispersity. The dispersity δ is also reported for each structure. From the analysis of the numerical results, there appears to be some correlation between the dispersity and the effective mechanical properties, however, due to the the limited dataset analysed here, further analyses are necessary to explore a broader range of dispersity levels and draw general conclusions.

Despite the level of dispersity in these systems, the mechanical properties still do not show considerable differences between different exemplars. As shown in Figure S6, the Poisson's ratios vary between -0.83 and -1.18, while the Young's moduli are also contained within a relatively restricted range. This means that the results obtained are still representative of the mechanical performance of this specific seed density, although it is probable that for the smallest seed density systems (i.e. $L_{RVE} = 2000$), more precise results with less standard deviation could be obtained if a larger number of seeds was used than $S = 45$.

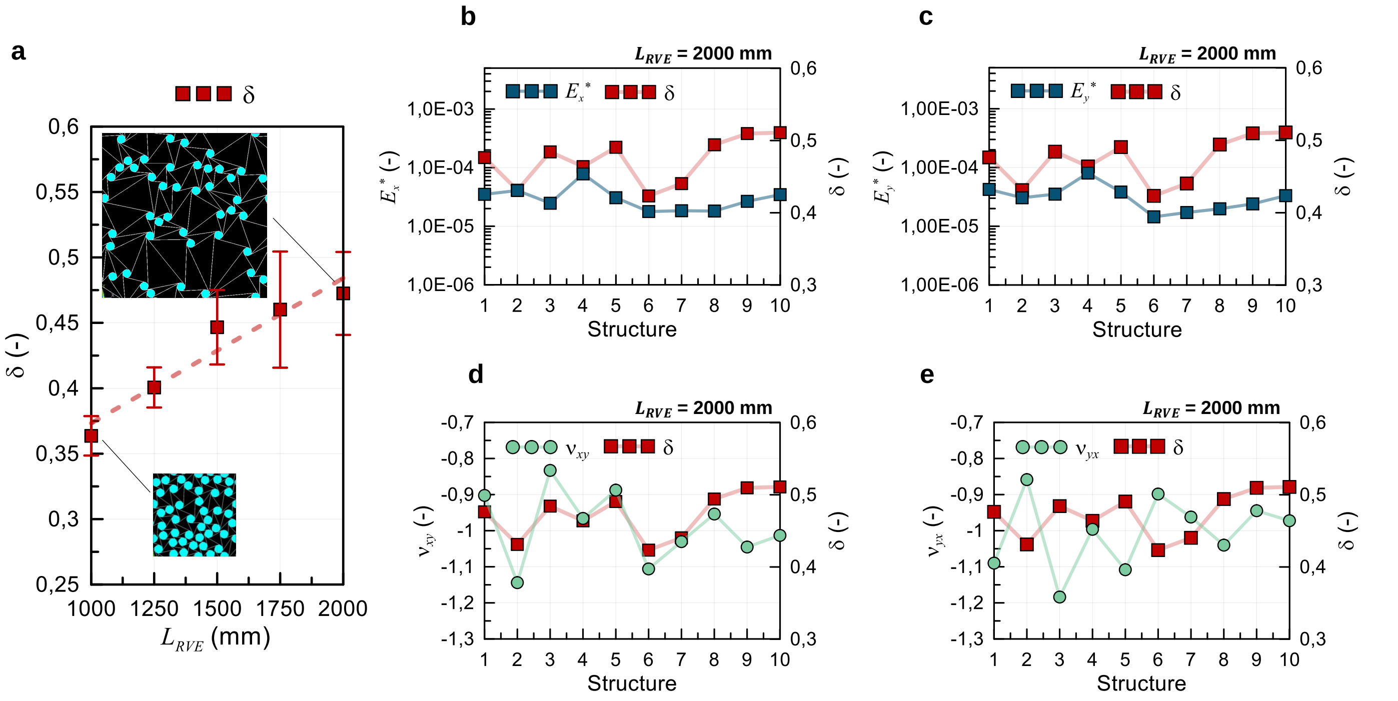

Fig. S6: Dispersity δ as a function of the dimension of the representative volume element L_{RVE} (a). Young's moduli E_x^* (b), E_x^* (c) and dispersity δ for structures with $L_{RVE} = 2000$ mm. Poisson's ratios ν_{xy} (d), ν_{yx} (e) and dispersity δ for structures with $L_{RVE} = 2000$ mm. Here, all the different structures are characterised by $D/t = 50$.

6 Influence of Geometric Parameters on Volume Fraction

The overall volume fraction, V_f, and, therefore density, of the disordered chiral system can be altered through variation of geometric parameters making up the system, i.e. the unit cell length (L_{RVE}), the ligament thickness (t) and the chiral node diameter (D). In Figure S7, the results are presented for a number of representative structures with only one paramter varied at a time whilst the other two remain constant. It is evident that the variation in L_{RVE}, hence, seed density, shows the largest change in volume fraction, while changes in ligament thickness yield the lowest. It is also worth noting that as shown in Figure 2 of the main manuscript, systems with large t values (i.e. values approaching D) tend to lose their auxetic behaviour. On the other hand, densifying the systems through variations of seed density has minimal effect on Poisson's ratio and high density systems with very negative Poisson's ratios can still be attained.

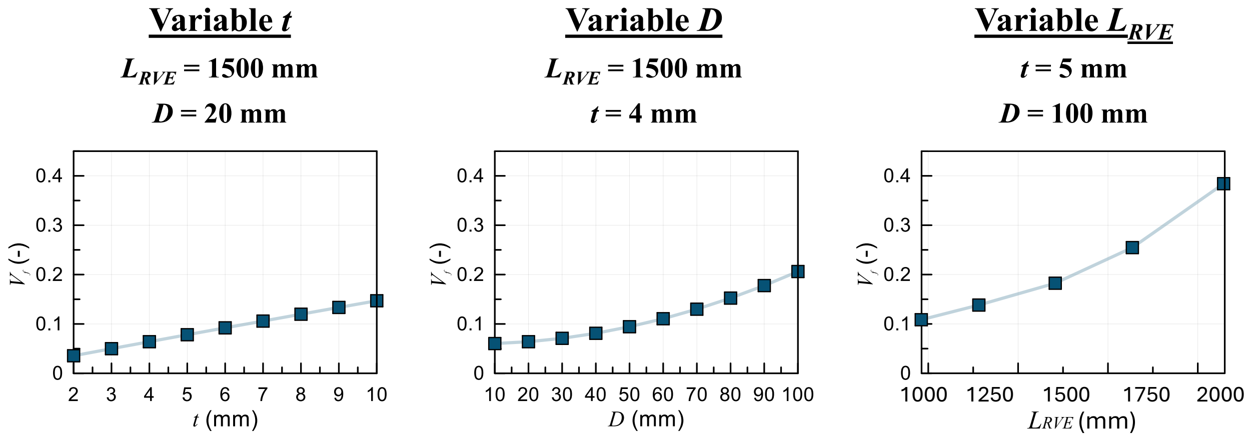

Fig. S7: Plots showing how Volume Fraction, V_f, varies with changes in t, D and L_{RVE}.

7 Stress-Strain Plot of 3D-Printed Polylactic Acid

Three dog-bones based on the ISO527 standard (Fig. S8a) were 3D-printed in the same orientation as the ligaments, i.e. orthogonal to the printing platform as shown in Fig. S8b, with 100% infill. The dog-bones were tested using a Galdabini tensile loading device with a 5kN loadcell at a constant tensile displacement velocity of 3 mm/min and the stress-strain plot obtained is presented in Fig. S8c. The Young's modulus obtained was 2200 MPa and the ultimate tensile strength was 35 MPa.

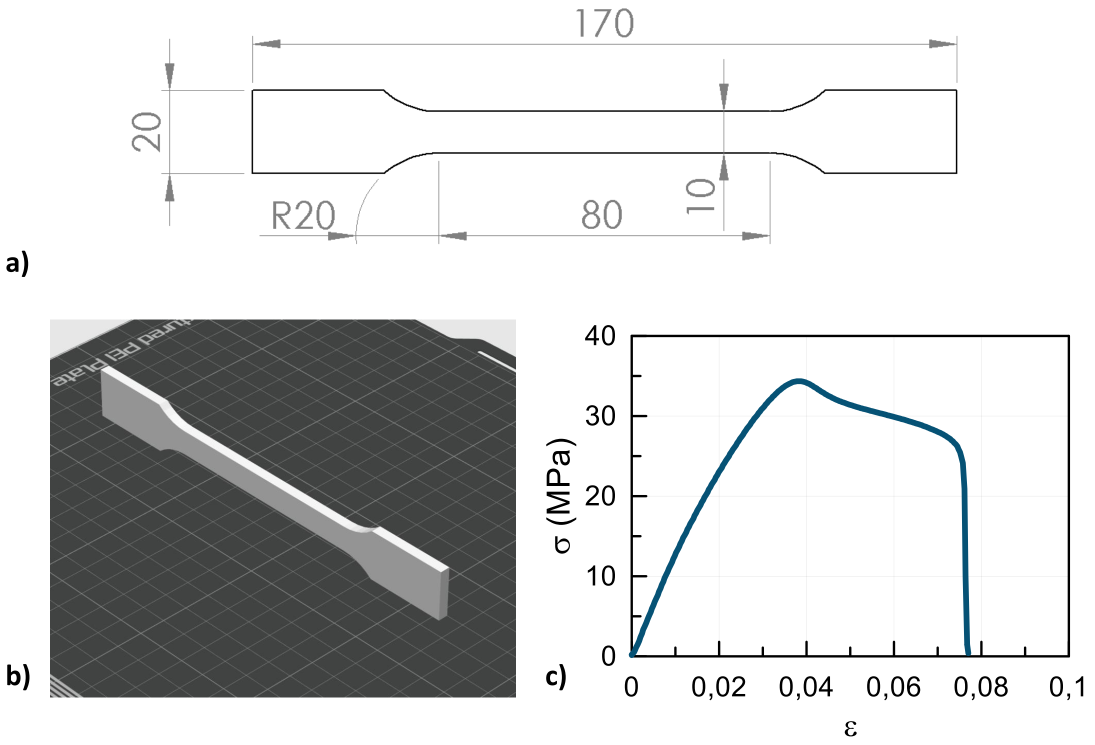

Fig. S8: Images showing a) the ISO527 dogbone, b) the printing orientation of the sample and c) the stress-strain plot obtained

8 Poisson's Ratio Measurement

As stated in the main manuscript, six samples (one ordered hexachiral and five disordered chiral honeycombs) were produced using additive manufacturing and tested under compressive loading conditions. The loading tests were recorded and the images analysed in order to determine the Poisson's ratio ν_{yx} of these systems. A set of virtual gauges were distributed along the transverse (x) and longitudinal (y) directions of the specimen (green and blue continuous lines in Fig. S9, respectively) and the lengths were measured. For each acquired frame, corresponding to a different applied deformation state, the engineering transverse and longitudinal strains, $\varepsilon_{x,i}$ and $\varepsilon_{y,i}$, were computed as the average values of the respective deformations of the virtual gauges.

In the case illustrated in Fig. S9, 11 gauges are present along the x-direction and 15 along the y-direction, thus ensuring a reliable measurement of the global Poisson's ratio of the system. All other tested specimens are equipped with a comparable number of virtual gauges. This approach was also utilised in the corresponding nonlinear Finite element simulation runs (which were carried out without PBCs and under exactly the same loading conditions as the experimental tests), thus allowing for an accurate comparison between the experimentally and numerically-obtained Poisson's ratios.

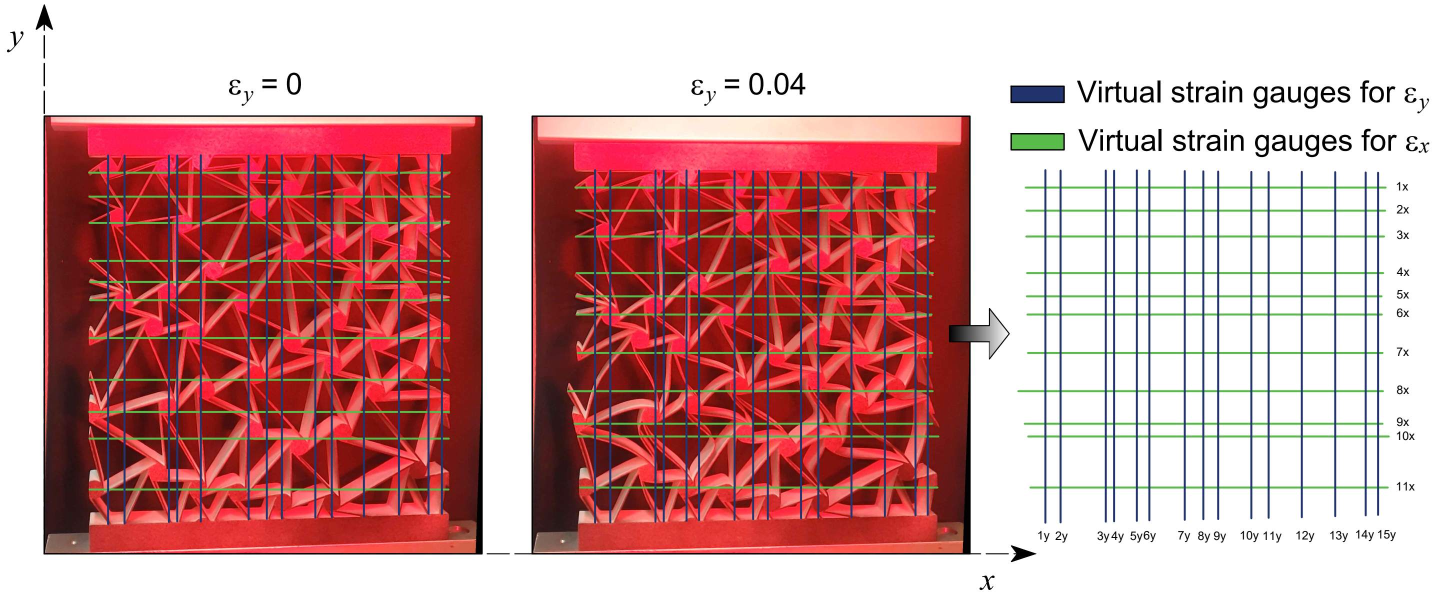

Fig. S9: View of the virtual strain gauges for determining the Poisson's ratio ν_{yx}, for $\varepsilon_y = 0$ and $\varepsilon_y = 0.04$.

9 Failure Mode Analysis

Once the Poisson's ratio was measured from the small-strain analysis of these systems, i.e. $\varepsilon_y = 0.04$, the failure modes of all six additively-manufactured samples were analysed under high strain conditions of up to $\varepsilon_y = 0.20$. The force-displacement curves are represented in Figure S10.

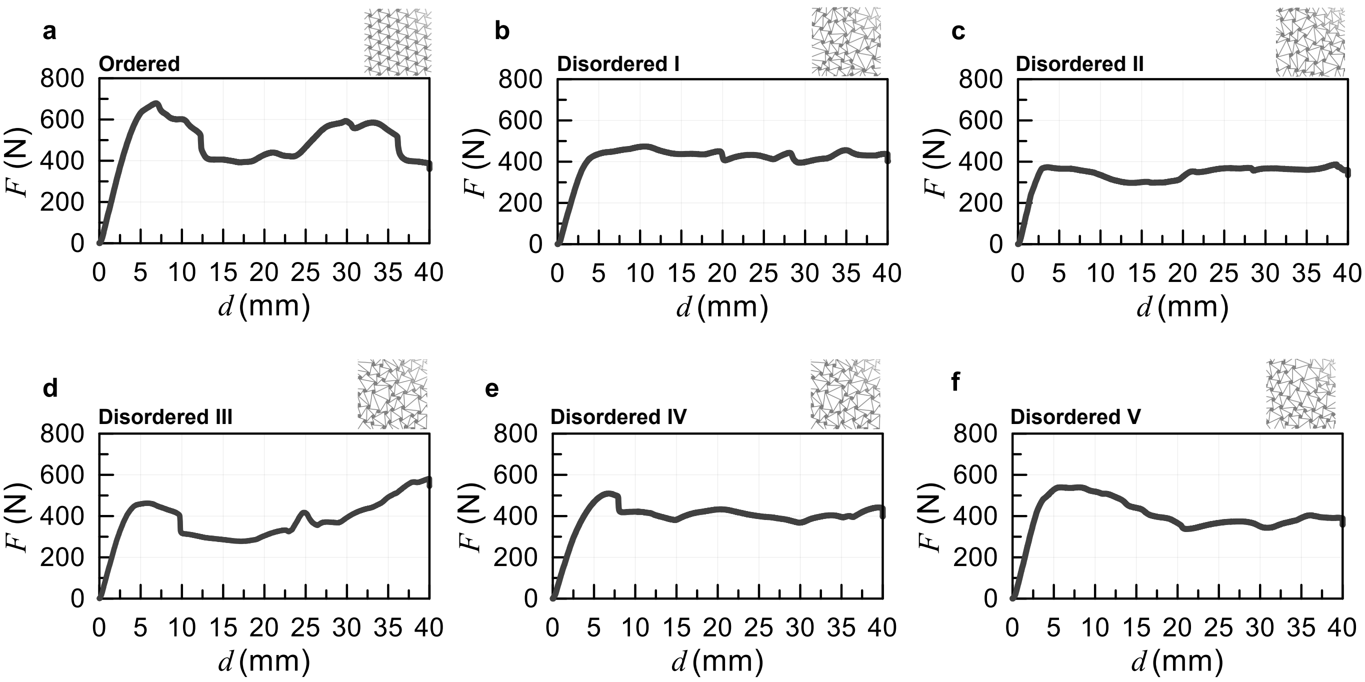

Fig. S10: Images showing different panels for all six samples at varying strains while indicating the evolving failure pathway throughout the systems. The first three structures are already presented in the main manuscript, although the panels here show different strain levels.

The analysis was carried out by measuring the deflection of ligaments and any ligaments which underwent deflection beyond a threshold value were marked as failed. The failure criterion utilised was based

on standard Euler-Bernoulli beam theory mechanics, specifically the standard model describing C-shaped flexure of ligaments [3]. This particular case was chosen on the basis that a preliminary visual analysis of the systems under loading revealed that C-shaped buckling of ligaments and highly asymmetric S-shaped flexure, which visually appears as a distorted C-shape, were by far the most dominant deformation modes observed in obviously failed ligaments (i.e. ligaments which remained permanently highly deformed post-compressive loading test). The equation for measuring the deflection, δ, of such a beam, is the following:

$$\delta = \frac{ML^2}{8EJ} \tag{S13}$$

where M is the applied flexural moment, L is the original ligament length, E is the constituent material Young's modulus and J is the second moment of inertia. The maximum localised stresses, σ_{max}, in such a beam would occur at the extremities and can be calculated as follows:

$$\sigma_{max} = \frac{My_{max}}{J} \tag{S14}$$

where y_{max} is the distance from the centroid of the beam to the extremity, in this case equal to half the cross-sectional beam thickness, $t/2$. Once the internal stress σ_{max} exceeds the ultimate tensile strength, S_u, then a ligament can be considered to have failed. Given that all ligaments have a rectangular cross-section with in-plane thickness t equal to 1 mm and out-of-plane thickness b equal to 20 mm and are made from PLA with a Young's modulus, E, of 2.2 GPa and an ultimate tensile strength, S_u, of 35 MPa, the failure criterion can be calculated in terms of the deflection/length squared ratio as follows:

$$\frac{\delta}{L^2} = \frac{S_u}{4Et} = 0.004 \text{ mm}^{-1} \tag{S15}$$

The deflections of the ligaments were measured for each frame and those which exceeded this threshold value of the ratio were marked sequentially per panel. As the strain increases, a distinct failure pathway becomes clearly visible. These pathways are marked on the final panel for each structure. Each panel analysed was chosen at an interval of *ca.* 3% strain.

References

[1] L. Mizzi, D. Attard, R. Gatt, K. K. Dudek, B. Ellul, J. N. Grima, *Engineering with Computers* **2021**, *37* 1765.

[2] R. Li, Q. Shao, E. Gao, Z. Liu, *Extreme Mechanics Letters* **2020**, *34* 100615.

[3] W. C. Young, R. G. Budynas, *Roark's Formulas for Stress and Strain*, McGraw-Hill, New York, seventh edition, **2002**.
